# Chiropractic spinal manipulative therapy for cervicogenic headache: a single-blinded, placebo, randomized controlled trial

**DOI:** 10.1186/s13104-017-2651-4

**Published:** 2017-07-24

**Authors:** Aleksander Chaibi, Heidi Knackstedt, Peter J. Tuchin, Michael Bjørn Russell

**Affiliations:** 1 Head and Neck Research Group, Research Centre, Akershus University Hospital, 1478 Lørenskog, Norway; 2 Institute of Clinical Medicine, Akershus University Hospital, University of Oslo, 1474 Nordbyhagen, Norway; 3 Department of Neurology, Innlandet Hospital Trust, 2418 Elverum, Norway; 4 Department of Chiropractic, Macquarie University, Sydney, NSW 2109 Australia

**Keywords:** Randomized controlled trial, Cervicogenic headache, Headache, Chiropractic, Spinal manipulative therapy, RCT

## Abstract

**Objective:**

Cervicogenic headache is a disabling headache where pharmacological management have limited effect. Thus, non-pharmacological management is warranted. Our objective was therefore to investigate the efficacy of chiropractic spinal manipulative therapy versus placebo (sham manipulation) and control (continued usual but non-manual management) for cervicogenic headache in a prospective 3-armed single-blinded, placebo, randomized controlled trial of 17 months’ duration.

**Results:**

Nineteen participants were equally randomized into the three groups, and 12 participants completed the randomized controlled trial. Headache frequency improved at all time points in the chiropractic spinal manipulative therapy and the placebo group. Headache index improved in the chiropractic spinal manipulative therapy group at all time points, while it improved at 6 and 12 months’ follow-up in the placebo group. The control group remained unchanged during the whole study period. Adverse events were few, mild and transient. Blinding was concealed throughout the RCT. Thus, our results suggest that manual-therapy might be a safe treatment option for participants with cervicogenic headache, but data need to be confirmed in a randomized controlled trial with sufficient sample size and statistical power.

*Trial registration* ClinicalTrials.gov identifier: NCT01687881, 11 September 2012

**Electronic supplementary material:**

The online version of this article (doi:10.1186/s13104-017-2651-4) contains supplementary material, which is available to authorized users.

## Introduction

Cervicogenic headache (CEH) is a secondary headache characterized by unilateral headache, and symptoms and signs of neck involvement [1, 2]. It is often worsened by neck movement, sustained awkward head position or external pressure over the upper cervical or occipital region on the symptomatic side [1, 2]. The prevalence of CEH varies from 1.0 to 4.6% in the general population depending on the diagnostic criteria [1, 3–6]. The efficacy of pharmacological management for CEH is poor and medication overuse is frequent [7]. Due to insufficient pharmacological treatment strategies, spinal manipulative therapy (SMT) has been recommended as a treatment option, despite the methodological shortcomings found in previous manual-therapy clinical trials [8, 9]; especially three-armed studies, including an active, a placebo, and a control group, have not previously been conducted, although this is recommended as the gold standard in RCTs.

The primary objective was to investigate the efficacy of chiropractic spinal manipulative therapy (CSMT) versus placebo (sham manipulation) and CSMT versus control (continued usual management but no manual-therapy were allowed during the trial period) in participants with CEH.

## Main text

### Methods

#### Design

This study was a prospective triple-armed, placebo RCT of 17 months’ duration with single blinded treatment and blinded outcome measures. The trial consisted of 1 month baseline, 3 months’ treatment with a total of 12 intervention sessions, and follow-up analysis at end of intervention and 3, 6, 12 months’. Participants were block randomized into: (a) CSMT, (b) placebo (sham manipulation), or (c) control (continued usual management but no manual-therapy were allowed during the trial period). The study design conforms the International Headache Society (IHS) and CONSORT [10–13]. The full trial protocol has been published previously and contains explicit details regarding the methodology [14].

#### Participants

Participants were recruited from September to October 2012 through the Akershus University Hospital and Innlandet Hospital Trust, Norway.

Eligible participants were between 18 and 70 years of age, diagnosed with a CEH by a neurologist, including at least three major criteria of the CHISG but not including occipital nerve blockage [1]. Exclusion criteria are described in details in the available protocol [14].

Eligible participants were invited to an interview and physical assessment by a chiropractor (AC) including meticulous investigation of the spinal column. Participants, who were randomized to CSMT or placebo, had a full spine radiographic examination prior to intervention commenced.

#### Intervention

The CSMT group received SMT using the Gonstead method, directed to spinal biomechanical dysfunction as diagnosed by standard chiropractic tests [15].

The placebo group received sham manipulation at the lateral edge of the scapula and/or the gluteal region [16].

The control group continued their usual pharmacological management without receiving manual intervention.

The interventions are described in details in the available trial protocol [14].

#### Blinding

After each treatment session, participants completed a de-blinding questionnaire, see protocol for details [16].

#### Outcome measures

Headache characteristics were recorded in the chiropractic in-depth interview followed by 1 month of validated diagnostic headache diary for baseline recordings [17], which was returned on a monthly basis [14].

The primary end-point was 25% reduction in number of headaches days per month (30 days/month) from baseline to end of intervention and 3, 6 and 12 months follow-up as compared to the placebo group and the control group respectively. Secondary end-points included a 25% improvement in headache duration, headache intensity and headache index (HI). HI was calculated as mean days with headache (30 days) × mean headache duration (hours per day) × mean intensity (0–10 NRS). End-points were based on IHS clinical guidelines [10, 11].

All adverse events (AEs) were recorded after each consultation in accordance with the CONSORT recommendations and IHS Task Force on AEs in migraine trials [12, 13].

#### Power calculation

The power was based on a recent group comparison study of topiramate [18]. A sample size of 20 patients was required in each group to detect a statistical significant average difference in reduction of 25% with 80% power, see protocol for details [14].

#### Statistical analysis

Data were recorded in MS Excel 2007. All analysis was performed by a blinded investigator using sealed serial number for each participant. Results for primary and secondary end-points are presented individually for each participant while mean change for each group are presented in figures. Mean HI and 95% confidence interval (CI) was calculated for baseline characteristics using SPSS v22.

#### Ethics and data security

All methods were carried out in accordance with the approved guidelines and regulations. Ethical details are available in the trial protocol [14].

### Results

Ninety-five participants with CEH diagnosed by a neurologist at Akershus University Hospital or Innlandet Hospital Trust, Norway were contacted. Twenty-one did not reply to the 1st or 2nd invitation letter. Among the 74 participants screened by telephone, 27 participants did not meet the inclusion criteria, i.e., nine (two men and seven women) had received CSMT within the last 12 months, four (one man and three women) had spinal radiculopathy, one woman had a dens fracture, one woman had a vertebral artery insufficiency, one woman had a cervical spine tumour, five (two men and three women) had headache remission, three (one man and two women) suffered depression, one woman had a brain tumour, one woman had insufficient Norwegian language skills, and one woman was pregnant.

Among the 47 eligible participants, 28 refrained to participate, i.e., due to time concerns (1 man and 11 women), physicians advised against participation (one man and two women), no faith in the treatment (one man and one woman), and unknown reason(s) (eight men and three women).

A total of 19 participants (5 men and 14 women) were randomized (Additional file 1). Three participants dropped out and four participants were excluded after randomization. Reasons for dropping out included, refrained to keep a headache diary (one woman), refrained to stop physiotherapy (one woman), and inclusion in an obesity rehabilitation program (one woman). Reasons for exclusion included, headache diaries not returned (two women), depression (one woman) and inflammatory disease (one man). Four participants were allocated to each of the three interventions. Baseline characteristic across the three groups were similar (Table 1).

**Table 1:** Baseline demographic and clinical characteristics

	Chiropractic spinal manipulative therapy	Sham manipulation (placebo)	Control group
Number of participants	4	4	4
Males	1	1	2
Females	3	3	2
Age ± SD (range)	36.0 ± 12.8 (26–54)	49.8 ± 12.3 (32–58)	48.0 ± 9.8 (39–61)
Duration (years with headache ± SD)	7.3 ± 3.3	8.5 ± 1.3	13.8 ± 10.4
Headache frequency (mean 30 days/month)	20.5 ± 9.9	25.5 ± 5.2	19.3 ± 7.9
Headache duration (mean hours/day)	19.5 ± 3.8	19.5 ± 7.7	19.3 ± 5.1
Headache intensity (mean 0–10 VAS)	6.0 ± 2.3	6.0 ± 0.7	7.0 ± 2.0
Co-morbid migraine	0/4	2/4	0/4
Primary end-point (1 month baseline)			
Headache frequency (mean 30 days/month)	20.0 ± 12.0	22.8 ± 12.3	19.0 ± 8.4
Secondary end-points (1 month baseline)			
Headache duration (mean hours/day)	9.6 ± 5.2	10.9 ± 3.3	9.9 ± 6.9
Headache intensity (mean 0–10 VAS)	6.6 ± 2.5	4.3 ± 1.7	6.3 ± 1.6
Headache index (HI) (mean frequency × duration × intensity)	956 95% CI 438 to 1475	1141 95% CI 283 to 1999	1635 95% CI −37 to 3307

#### Outcome measures

Individual changes in headache frequency, duration, intensity and HI from baseline to post-treatment and 3, 6 and 12 months follow-up are presented in Table 2.

**Table 2:** Mean changes for each participant from baseline to post-treatment and 3, 6 and 12-months follow-up

	Chiropractic spinal manipulative therapy (CSMT)				Sham manipulation (placebo)				Control group			
Baseline	I	II	III	IV	V	VI	VII	VIII	IX	X	XI	XII
Headache frequency	30	14	30	6	30	12	19	30	13	12	21	30
Headache duration	15.4	4.6	5.7	12.5	13.3	6.4	10.6	13.3	7.3	1.4	15.1	15.8
Headache intensity	3.7	9.4	5.5	7.6	5.9	3.7	5.4	2.1	4.4	5.6	7.2	7.9
Headache index	1709	605	941	570	2354	284	1088	838	418	94	2283	3745
Post-treatment												
Headache frequency	30 (0.0)	11 (21.4)	12 (60.0)	3 (100.0)	30 (0.0)	6 (50.0)	22 (15.8)	0 (100.0)	13 (0.0)	14 (16.7)	20 (4.8)	29 (3.3)
Headache duration	7.0 (54.5)	10.2 (121.7)	2.7 (52.6)	7.8 (37.6)	14.6 (9.8)	4.2 (34.4)	10.6 (0.0)	0 (100.0)	4.1 (43.8)	1.0 (28.6)	13.3 (11.9)	15.4 (2.5)
Headache intensity	1.2 (67.6)	9.6 (2.1)	2.2 (60.0)	4.2 (44.7)	6.1 (3.4)	4.8 (29.7)	6.7 (24.1)	0 (100.0)	5.1 (15.9)	4.1 (26.8)	5.9 (18.1)	8.6 (8.9)
Headache index	252 (85.3)	1077 (78.0)	71 (92.5)	98 (82.8)	2672 (13.3)	121 (57.4)	1562 (43.6)	0 (100.0)	272 (34.9)	57 (39.4)	1569 (31.3)	3841 (2.6)
3-months follow-up												
Headache frequency	30 (0.0)	5 (64.3)	8 (73.3)	11 (83.3)	30 (0.0)	7 (41.7)	20 (5.3)	6 (80.0)	17 (30.8)	2 (83.3)	25 (19.0)	30 (0.0)
Headache duration	11.0 (28.6)	6.2 (34.8)	1.9 (66.7)	12.6 (0.8)	12.9 (3.0)	6.8 (6.2)	13.1 (23.6)	4.2 (68.4)	6.1 (16.4)	5.3 (278.6)	15.1 (0.0)	15.0 (5.1)
Headache intensity	2.3 (37.8)	7.5 (20.2)	1.9 (65.4)	6.8 (10.5)	4.9 (16.9)	5.4 (45.9)	8.1 (50.0)	1.0 (52.4)	4.8 (9.1)	1.0 (82.1)	6.8 (5.6)	8.1 (2.5)
Headache index	759 (55.6)	233 (61.5)	29 (96.9)	942 (65.3)	1896 (19.5)	257 (9.5)	2122 (95.0)	25 (97.0)	498 (19.1)	11 (88.3)	2567 (12.4)	3645 (2.7)
6-months follow-up												
Headache frequency	30 (0.0)	5 (64.3)	9 (70.0)	13 (116.7)	30 (0.0)	6 (50.0)	17 (10.5)	8 (73.3)	13 (0.0)	14 (16.7)	26 (23.8)	30 (0.0)
Headache duration	11.0 (28.6)	5.4 (17.4)	2.3 (59.6)	12.8 (2.4)	9.4 (29.3)	11.2 (75.0)	10.8 (1.9)	13.9 (4.5)	3.9 (46.6)	1.0 (28.6)	15.8 (4.6)	15.0 (5.1)
Headache intensity	1.7 (54.1)	8.8 (6.4)	1.4 (74.5)	7.2 (5.3)	3.2 (45.8)	6.3 (70.3)	6.9 (27.8)	0.9 (57.1)	4.7 (6.8)	4.1 (26.8)	6.9 (4.2)	8.5 (7.6)
Headache index	561 (67.2)	238 (60.7)	29 (96.9)	1198 (110.2)	902 (61.7)	423 (48.9)	1267 (16.5)	100 (88.1)	238 (43.1)	57 (39.4)	2835 (24.2)	3825 (2.1)
12-months follow-up												
Headache frequency	30 (0.0)	4 (71.4)	4 (86.7)	8 (33.3)	30 (0.0)	2 (83.3)	19 (0.0)	8 (73.3)	15 (15.4)	20 (66.7)	28 (33.3)	30 (0.0)
Headache duration	12.1 (21.4)	5.6 (21.7)	2.5 (56.1)	12.5 (0.0)	13.9 (4.5)	7.5 (17.2)	8.7 (17.9)	8.6 (35.3)	10.7 (46.6)	1.3 (7.1)	15.7 (4.0)	15.0 (5.1)
Headache intensity	1.5 (59.5)	9.4 (0.0)	9.4 (70.9)	7.9 (3.9)	4.6 (22.0)	4.9 (32.4)	6.3 (16.7)	1.1 (47.6)	6.0 (36.4)	4.6 (17.9)	5.6 (22.2)	8.1 (2.5)
Headache index	545 (68.1)	211 (65.1)	15 (98.4)	790 (38.6)	1918 (18.5)	74 (73.9)	1041 (4.3)	76 (90.9)	963 (130.4)	120 (27.7)	2462 (7.8)	3645 (2.7)

**Fig. 1:**
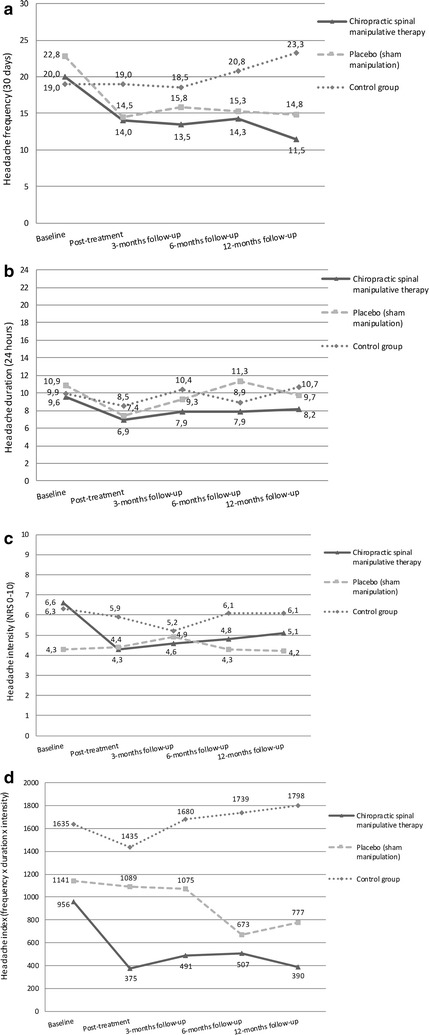
**a**–**d** Mean changes from baseline to follow-up for primary and secondary end-points

Mean change in primary and secondary end-points for each group are presented in Fig. 1a–d.

The main effect of the treatment was on headache frequency in both the CSMT and the placebo group, an effect that was maintained at follow-up (Fig. 1a).

Headache index improved in the CSMT group at all time points while it improved at 6 and 12 months follow-up in the placebo group.

The control group remained unchanged during the whole study period (Fig. 1a–d).

#### Blinding

The four participants who received CSMT believed they received it with a mean 9.1 certainty, whereas the four participants who received placebo believed they received active CSMT with a mean 6.4 certainty.

#### Adverse effects

Mild transient AEs were local tenderness and tiredness on the treatment day in both the CSMT and the placebo group. No severe or serious adverse events were reported in the study.

### Discussion

To our knowledge, this is the first triple-armed chiropractic manual-therapy RCT to include a placebo and a control group for participants with CEH. The study is also the first to successfully maintain blinding throughout a full length intervention period. Our main results demonstrate reduction in headache frequency and HI in the CSMT and the placebo group, an effect that lasted at follow-up, while the control group was unchanged throughout the RCT.

#### Methodological considerations

As compared to previous manual-therapy RCTs, our participants were selected based on more rigorous criteria for CEH and the diagnosis was confirmed by a neurologist experienced in headache disorders [9]. The exclusion criteria i.e., depression, chiropractic treatment within the previous 12 months and pregnancy, excluded 36% of the participants. Furthermore, rigorous RCT rules limiting participants to omit from any manual therapies throughout the 12 months’ follow-up period was necessary to obtain a homogenous sample population, avoid type-II errors, and enable a successful blinding in the placebo group.

Similar to other studies of CEH, and considering that the prevalence of CEH is 0.17% in the general Norwegian population [7], we experienced severe challenges in recruiting and maintaining participants in the trial. Thus, we can only present descriptive data. Although a cross-over design would have strengthen the power, several limitations exists with this design, i.e., (a) longer intervention period extended by a wash-out period; (b) the ethical concerns regarding switching from a successful treatment to placebo; (c) reports of AEs could possible unmask the blinding. It was also surprising that 38% declined to participate, primarily due to time concerns which might indicate that their CEH might be less severe or lack of enthusiasm due to previous therapy failures [19].

The strength of our RCT includes diagnosis and intervention by a single experienced chiropractor which contribute to a strong internal validity. Prospective headache diaries give near exact measurements. The HI has despite the lack of consensus been recommended as a measurement outcome to give an indication of the total level of suffering [11].

#### Results discussion

The results in this trial are similar to previous results reported in reviews on SMT for CEH [9, 20]. A Danish RCT found headache intensity to reduce by 36 and 22% in the SMT group at post treatment and 1 week follow-up respectively as compared to the soft tissue treatment group [21]. An Australian RCT with high methodological quality reported 71% of the participants having >50% reduction in headache frequency while 33% reported a 100% improvement in the SMT group [22]. Two American RCTs reported a mean reduction of 43, 29 and 40% reduction in headache intensity at 4, 12 and 24 weeks follow-up respectively in the SMT group, while mean headache frequency similarly reduced by 49, 34 and 52% respectively [23, 24].

The placebo effect is known to be high in headache RCTs and assumed similar high for non-pharmacological clinical trials [25–27]. The placebo effect for headache frequency was high in our RCT, while HI improved first at 6 months follow-up in the placebo group. One should therefore not disregard the fact that the effect shown in our RCT could be a placebo effect.

A confounding factor for which no improvement in days with headache was omitted for one participant in the CSMT group, i.e., participant one; and two participants in the placebo group, i.e., participant five and seven according to their diaries, was the fact that they probably suffered medication overuse headache [2].

It is not uncommon that participants receiving placebo intervention report similar AEs as seen in the active intervention, likely produced by expectations [28, 29]. AEs following placebo administration in pharmacological clinical trials for primary headache disorders has been reported as high as 43% [30], often related to the study information letter, the informed consent and attitude towards participants. Considering our success in concealing the blinding, the latter fact seems unlikely. However, the true nature of the nocebo effects cannot be identified from our three-armed RCT since the control group was included to quantify the placebo effect, and AEs was not monitored in the control group.

Finally, one participant in the placebo group i.e., participant eight, experienced daily headache frequency at baseline and no headache post-treatment, which slightly increased at 3, 6 and 12 months follow-up. This certainly influenced the results in the placebo group, and the complete resolution of the headache at post treatment stay in contrast to the other participants, and one can speculate whether this is a real phenomenon or an issue of non-compliance when returning the headache diary.

Given the limited effect of pharmacological management for CEH, this study adds knowledge to previous observed effects from SMT [9]. Unfortunately, the low sample size limits our conclusions; replications of this trial with a substantial increased sample size which follows the recommended IHS clinical trial guidelines are needed to confirm the results [10, 11]. Our attempt to blind participants, in order to establish a placebo group in a manual-therapy RCT was our most successful result.

## Limitations

Small sample size enforced us to present descriptive data and limits our conclusions.

## Additional file

**Additional file 1.** Participants flow diagram.

## Abbreviations

AE: adverse event
CEH: cervicogenic headache
CI: confidence interval
CSMT: chiropractic spinal manipulative therapy
HI: headache index
HVLA: high velocity low amplitude
IHS: International Headache Society
MOH: medication overuse headache
RCT: randomized controlled trial
SMT: spinal manipulative therapy
