# Supplementary figures and images for: Chiropractic spinal manipulative therapy for cervicogenic headache: a single-blinded, placebo, randomized controlled trial
Source: BMC Res Notes. 2017 Jul 24;10:310. doi: 10.1186/s13104-017-2651-4 (PMC5525198; doi:10.1186/s13104-017-2651-4)

## Participants flow diagram

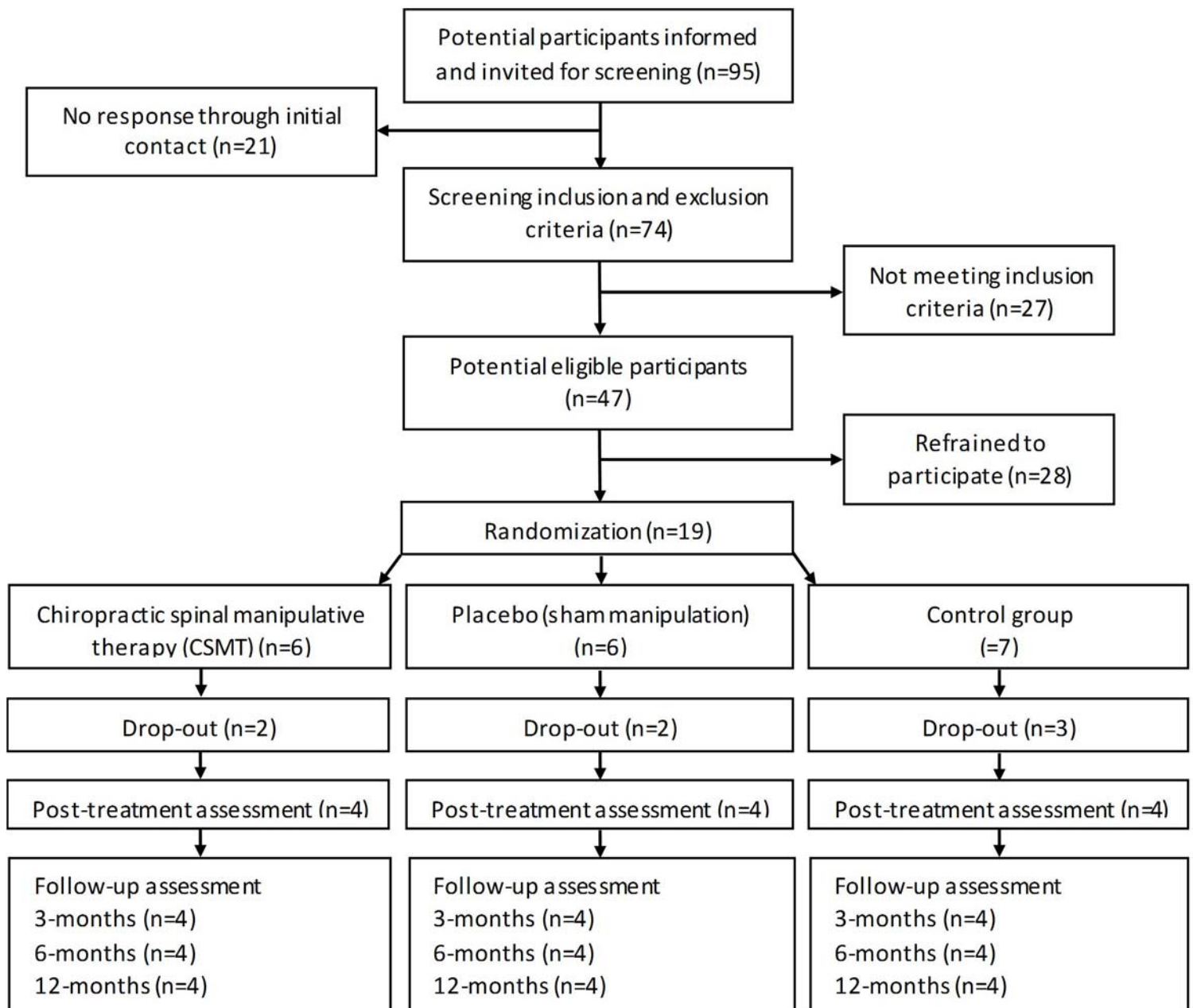

Supplement: Supplementary file 1 — Additional file 1. Participants flow diagram. [file 13104_2017_2651_MOESM1_ESM.pdf]
